# Preference, performance, and chemical defense in an endangered butterfly using novel and ancestral host plants

**DOI:** 10.1038/s41598-020-80413-y

**Published:** 2021-01-14

**Authors:** Nathan L. Haan, M. Deane Bowers, Jonathan D. Bakker

**Affiliations:** 1 School of Environmental and Forest Sciences, University of Washington, Seattle, Box 354115, Seattle, WA 98195 USA; 2 Ecology and Evolutionary Biology and Museum of Natural History, University of Colorado at Boulder, UCB 334, Boulder, CO 80309 USA; 3 Department of Entomology, Michigan State University, East Lansing, USA

**Keywords:** Community ecology, Conservation biology, Evolutionary ecology

## Abstract

Adoption of novel host plants by herbivorous insects can require new adaptations and may entail loss of adaptation to ancestral hosts. We examined relationships between an endangered subspecies of the butterfly *Euphydryas editha* (Taylor’s checkerspot) and three host plant species. Two of the hosts (*Castilleja hispida, Castilleja levisecta*) were used ancestrally while the other, *Plantago lanceolata*, is exotic and was adopted more recently. We measured oviposition preference, neonate preference, larval growth, and secondary chemical uptake on all three hosts. Adult females readily laid eggs on all hosts but favored *Plantago* and tended to avoid *C. levisecta.* Oviposition preference changed over time. Neonates had no preference among host species, but consistently chose bracts over leaves within both *Castilleja* species. Larvae developed successfully on all species and grew to similar size on all of them unless they ate only *Castilleja* leaves (rather than bracts) which limited their growth. Diet strongly influenced secondary chemical uptake by larvae. Larvae that ate *Plantago* or *C. hispida* leaves contained the highest concentrations of iridoid glycosides, and iridoid glycoside composition varied with host species and tissue type. Despite having largely switched to a novel exotic host and generally performing better on it, this population has retained breadth in preference and ability to use other hosts.

## Introduction

The spread of exotic organisms results in novel species interactions that can produce evolutionary changes^1^. One increasingly common set of interactions occurs when native herbivorous insects encounter and utilize exotic plant species. From the perspective of a specialist herbivore, there can be a range of ecological and evolutionary consequences for adopting a new host. These consequences can include changes to abundance, range, morphology, phenology, voltinism, diet preference, and even the development of new host-specific ecotypes^1–6^.

Novel interactions between herbivores and plants, and their evolutionary consequences, can intersect with and complicate conservation efforts. Conservation of herbivorous insects, such as endangered butterflies, often hinges on understanding relationships with host plants, as host suitability varies within and among plant species and suitable hosts can be rare^7^. When butterflies of conservation concern begin ovipositing on an exotic species, is the new resource an asset or a liability? Some studies have demonstrated an immediate risk of an ecological trap^8^, in which eggs are laid on the exotic species but survival is low^9,10^. Others have shown that the butterfly successfully adopts its new host, either by adapting to it or because of ecological fitting, as when the new host is suitable given the butterfly’s previous adaptations^11^. However, when a new host is successfully adopted it can result in lost adaptation to ancestral hosts, both in terms of adult preference and larval ability to develop^4,12^. In such circumstances, it is possible for a longer-term trap to arise in which a population adapts to selective pressure from the new host, but in so doing loses its ability to persist over the longer term because it becomes a poor fit for its former host^12^.

An iconic group of organisms that exist at the intersection of these issues and exemplify their complexity are checkerspots in the genus *Euphydryas* (Lepidoptera: Nymphalidae). Several taxa within this group have incorporated the exotic species *Plantago lanceolata* into their diet^13–15^. Probably the best studied checkerspot in North America is Edith’s checkerspot, *E. editha*, which is composed of multiple subspecies, several of which have declined and are listed under the US Endangered Species Act^16–18^. These butterflies and their relatives have been models for understanding population ecology, rapid evolution of host plant affiliations, and chemical ecology^19^. Ironically, despite being heavily researched, these organisms can be perplexing to conserve; variability in host plant affiliations within and among subspecies and populations complicates recovery efforts.

Checkerspots affiliate with several plant taxa that produce iridoid glycosides (hereafter IGs). At the species level, *E. editha* is not very host-specific and uses a wide range of IG-producing plants, but individual populations are often monophagous with host- and location-specific adaptations^19^. Host plant preference in this species is heritable^20^ and can evolve quickly in response to shifts in availability of host resources^12,21^. Divergent specialization can also occur within populations, such that subgroups within populations differ in adult oviposition preference for, and larval performance on, one host or another^20^.

The iridoid glycosides found in checkerspot host plants likely serve as an oviposition cue^22,23^, and larvae sequester them in their hemolymph, making them unpalatable to predators through the adult stage^24–26^. Herbivores that sequester IGs from plants take up different sets of compounds, in varying concentrations, depending on which plant species they eat^26^ or according to the tissue type or ontogenetic stage consumed^27^. These differences in what and how much they sequester can in turn influence their interactions with higher trophic levels^24,28^.

**Figure 1:**
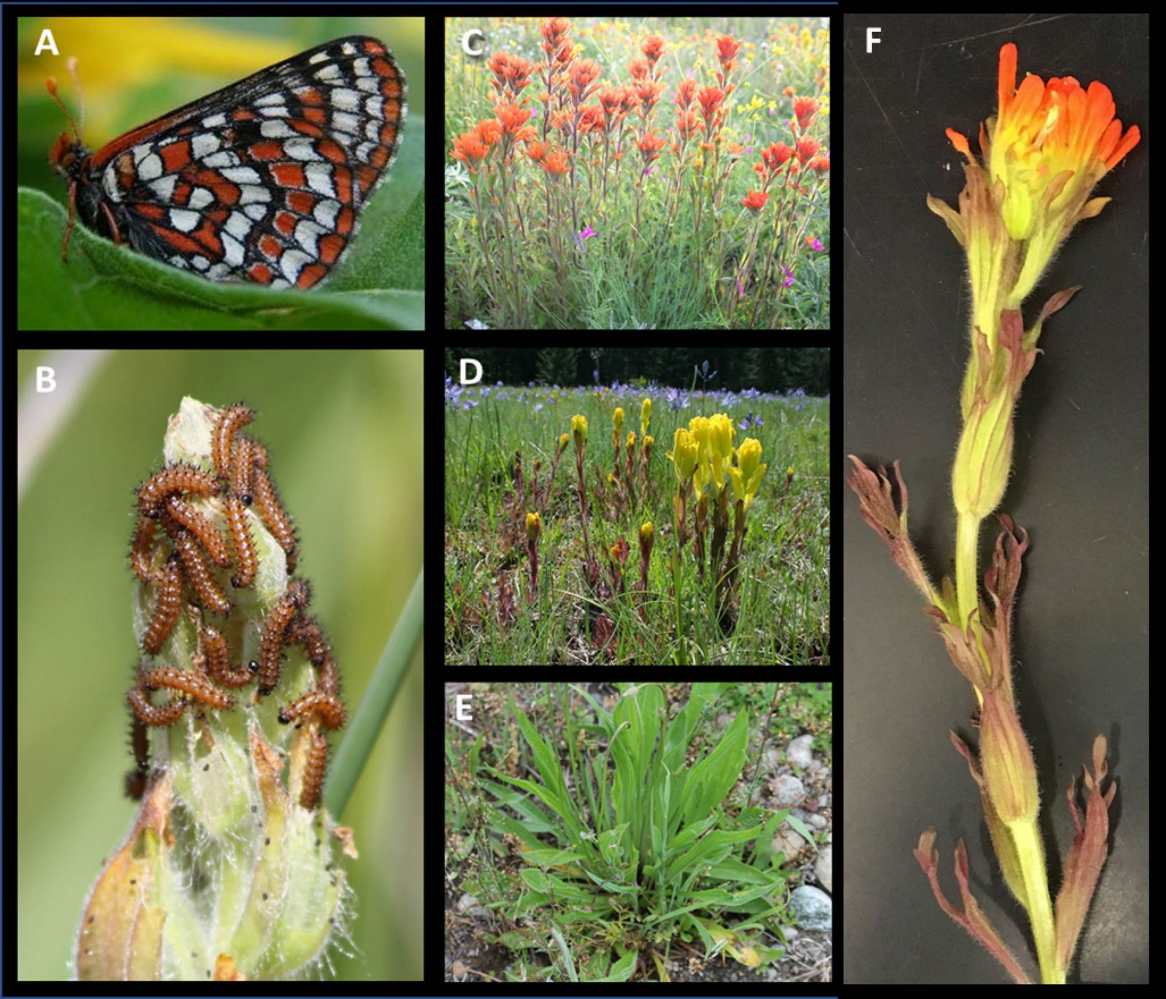
Taylor’s checkerspot (**A**) adult and (**B**) larvae, and host plants (**C**) *Castilleja hispida,* (**D**) *Castilleja levisecta*, and (**E**) *Plantago lanceolata.* (**F**) Closeup of *C. hispida* illustrating the contrast between leaf and bract tissues. Photos: N. Haan.

Taylor’s checkerspot (*Euphydryas editha taylori*; Fig. 1A,B) is one of the endangered subspecies of *E. editha* and is endemic to grasslands of the Pacific Northwest in North America. It was listed under the US Endangered Species Act in 2013^18^. Its interactions with host plants are poorly understood, with knowledge gaps delaying recovery efforts and hampering grassland conservation practices in the region^29^. Formerly quite common, Taylor’s checkerspot existed in metapopulations made up of dense, sedentary colonies occurring in grasslands. At the time of listing, it had declined to only 10 populations, mostly in Washington state, USA. Its decline is thought to be a result of habitat loss, disruption of historic fire regimes, and lost metapopulation dynamics as individual populations disappeared^29,30^.

Historically, Taylor’s checkerspot is thought to have mostly used the paintbrushes *Castilleja hispida* and *Castilleja levisecta* (Orobanchaceae) for oviposition (Fig. 1C,D)*,* although at later stages in larval development it can also eat winter annuals such as *Plectritis congesta* (Valerianaceae) and *Collinsia* spp. (Plantaginaceae)^29,30^. Most remaining Taylor’s checkerspot populations have incorporated *Plantago lanceolata* (hereafter, *Plantago*; Plantaginaceae) into their diet (Fig. 1E). *Plantago* is thought to have been introduced to North America about 200 years ago^31^. It produces IGs and is readily available since it is ubiquitous in human-disturbed habitats. In addition to being generally more abundant than native hosts, its phenology is also more favorable for larvae as *Castilleja* spp. can senesce quickly in dry conditions^32^. While *Plantago* cannot have been an ancestral host plant for Taylor’s checkerspot*,* it was the first documented host and some populations used it as early as the late nineteenth century^33^.

This study focuses on the last naturally occurring Taylor’s checkerspot population in lowland habitats of southwest Washington State, USA. The specific history of host plant use by this population is unknown, but when it was discovered in 2003 the butterflies were reliant on *Plantago.* A very small number of *C. hispida* were also present at the site and may have been used, but these were only sufficient for < 1% of the population to use for oviposition. In recent years, more *C. hispida* was introduced to the site in low densities and larvae now feed on both species (M. Linders, Washington Department of Fish and Wildlife, personal communication). Recovery efforts involve a captive rear-and-release program in which offspring of individuals sourced from this population are reared (on *Plantago*) and released at other potentially suitable sites in the region. These sites vary in several respects, including abundance of potential hosts (*C. hispida, C. levisecta, Plantago*), and therefore, from a conservation standpoint, it is important to know whether Taylor’s checkerspot has retained its ability to use other host species or whether it is now better adapted to *Plantago*.

Host plant affinities are multidimensional and can include female decisions of where to oviposit, innate neonate preference for where to forage, and larval performance when feeding on one host plant or another. Previous work with this population suggests that females prefer to oviposit on either *Castilleja* species over *Plantago*^34^, but that survival rates of early-instar larvae in the field are highest on *Plantago,* intermediate on *C. hispida*, and lowest on *C. levisecta*^32^*. Plantago* and *Castilleja* spp. also have contrasting architectures, leading to differences in resource distribution and larval feeding behavior: *Plantago* forms a low rosette, while both *Castilleja* species are oriented vertically with leaves toward the base and, later in the spring, flowers subtended by showy bracts toward the top (Fig. 1F). We have generally observed that Taylor’s checkerspot lay their eggs toward the base of *Castilleja* plants, but larvae move toward the apex and are often seen eating bracts and flowers rather than the lower leaves where eggs are placed (Haan, pers. obs.). The bracts and flowers are younger than the lower leaves and may also differ chemically; in other *Castilleja* species there are strong differences among tissues in the presence and abundance of IG compounds^35^.

We used a laboratory study to examine how adopting a novel host may have influenced host plant preference and performance within this population of Taylor’s checkerspot. Although it has shifted largely to feeding on *Plantago*, we wanted to know if it has retained a preference for and ability to develop on its ancestral *Castilleja* hosts. We also tested for within-population differences in host plant specialization, which would be evidenced by correlations between maternal preference, neonate preference, and larval performance on a given species. Our specific objectives were to: (1) quantify oviposition preference among the three host species; (2) quantify neonate preference among the three host species and between bract and leaf tissue within each *Castilleja* species*;* (3) compare growth among larvae raised on each species and between larvae that fed on different *Castilleja* tissue types; (4) test if secondary chemical uptake depends on which species and/or tissue type larvae eat; (5) test for correlations between maternal and neonate preference for each host plant species and whether either type of preference for a given species is correlated with mass gain on that species.

## Methods

### Butterfly collection and rearing

Butterflies used in this study were second-generation captives originating from the last extant population of *E. e. taylori* in the South Puget Sound region, on an active artillery impact area at Joint Base Lewis-McChord, Washington, USA. At this site, butterflies oviposit and larvae feed primarily on *Plantago,* but occasionally on *C. hispida* as well. Butterflies were collected from the field in 2016 and allowed to oviposit in the lab; the eggs were then deployed for a field study^32^. In late winter 2017, we collected surviving larvae from the field study after they emerged from diapause. These larvae fed on *Plantago* during early instars in the field, and we continued to feed them this species until pupation. In the lab, larvae were reared in containers with other members of their sibling group. After eclosion, male and female butterflies were separated, and females (one per sibling group) were mated to non-sibling males using methods described by Barclay et al.^36^. They were fed a 3:1 water:honey mixture daily. Butterflies used in this study were collected and handled in accordance with a USFWS permit.

### Objective 1: oviposition choice trials

We tested oviposition preference of 29 mated *E. e. taylori* females (matrilines) in 2017*.* We created three-way choice trials by enclosing females with mesh screening on 2.5 L pots with all three host plant species growing in each pot. Mesh was tented with bamboo stakes and held to the pot with a rubber band. The host plants were grown from seed in greenhouses at the University of Washington Center for Urban Horticulture the previous year, then stored outdoors over winter and transplanted so all three species shared a pot (n = 42 pots) in spring 2017. The growing medium in each pot was covered in a layer of pea gravel. At the beginning of each choice trial, a mated female butterfly was placed on a cotton swab soaked in dilute honey solution and introduced to a screened-in pot while it was feeding, with the swab placed upright between the three plants. Pots were placed under full spectrum lighting in the lab, or when possible, in direct sunlight outdoors, until either one day had elapsed or we observed that the butterfly had laid eggs. At this point the butterfly was fed and introduced to a new enclosure using the same methods. After each choice trial, we inspected all leaves of all plants in the pot and removed leaves where eggs had been laid. Pots were re-cycled for trials with other individuals until ~ 20% of leaves had been removed from a plant. In all, 122 oviposition choice trials were made (mean = 4.2 choices per individual, range = 1–8). We quantified preference as the number of eggs laid. Checkerspots oviposit in clutches of varying size and preference can also be quantified in terms of number of egg clusters laid rather than individual eggs. We focus on number of eggs laid because we suspected the number of eggs per cluster would be confounded with plant species due to differences in architecture (*Castilleja* have smaller leaves which could lead butterflies to lay fewer eggs per cluster and more clusters overall). However, we also report the proportion of clusters laid on each species and results were qualitatively very similar (see “Results”).

Eggs were stored in 60 mL plastic containers lined with paper towel and with perforated lids until they neared hatching (indicated by darkening). At this time, clusters were separated into smaller groups with a fine-tipped paintbrush so they could be allocated to either neonate choice trials (Objective 2) or no-choice feeding trials (Objectives 3 and 4).

We analyzed oviposition preference at both the population and individual level. For the population-level assessment, we assigned each butterfly to the host species it laid the most eggs on, and used a chi-square test to evaluate whether the number of individuals preferring each species differed. For the individual-level assessment, we began by visually comparing the data for overall oviposition patterns among matrilines and then tested if preference among hosts changed over time using binomial generalized linear mixed models (GLMMs) with each oviposition trial as a replicate (n = 122). We included random effects for the matriline and for the pot used in the trial since individuals made multiple choices and pots were re-cycled. We tested each plant species separately. For each species, the response was the number of eggs laid on that species compared to the other species. We carried out these and all other statistical analyses in R version 3.6.2^37^, and used the *lme4*^38^ package for all mixed models.

### Objective 2: neonate choice trials

Twenty-one of the 29 butterflies produced enough viable eggs to be used in neonate choice trials (3 sets of trials x minimum of 3 eggs per trial = 9 eggs required). For each matriline, we placed groups of 3–6 darkened eggs in choice trial arenas (3 × 5 cm plastic cells). We used groups of eggs rather than individual eggs because larvae are gregarious during early instars. We cut 6 mm diameter leaf discs from each plant species (field collected, see below) using an office hole puncher and arranged the discs around each cluster of eggs so that newly hatched larvae had equal access to all discs. Discs and eggs were placed on beds of moistened paper towel to keep them from desiccating. We rotated the spatial arrangement of leaf discs systematically to avoid spatial or directional effects.

We conducted three sets of choice trials (n = 21 for each). In one set of trials, neonates chose between leaves of all three species; in the other two they chose between leaves and bracts from the same *C. hispida* or *C. levisecta* stem. After hatching, larvae fed for ~ 24 h or until we observed that they consumed a whole leaf disc, at which point we ended the trial. We photographed each leaf disc and assessed the area eaten using ImageJ^39^.

We tested whether neonate preference, expressed as the proportion of total leaf area consumed, varied among host species using a GLMM with a binomial distribution and matriline as a random effect. This model had a singular fit so we refit it as a GLM with matriline included as the first fixed effect. Finally, we tested whether neonates ate more tissue from lower leaves or from bracts. These tests were conducted separately for each *Castilleja* species, using paired t-tests.

### Objective 3: larval growth

Twenty matrilines produced enough viable eggs to be used in no-choice feeding trials (5 treatments x minimum of 4 larvae per treatment = 20 larvae required for a matriline to appear in all treatments). We assigned freshly-hatched sibling neonates from each matriline to five feeding treatments. Larvae were fed ad libitum diets of *P. lanceolata* leaves*, C. hispida* leaves, *C. hispida* bracts, *C. levisecta* leaves, or *C. levisecta* bracts. We cut *Castilleja* stems in half so members of the same matriline ate the same *Castilleja* stems but were restricted to either leaf or bract. Three of the 20 matrilines produced enough larvae to be included in only three of the five treatments, so for these we omitted the treatments with *Castilleja* leaves because in the field we generally observe larvae eating bracts. Numbers of individual larvae per replicate varied from 4 to 10 among matrilines depending on availability, but group sizes were equal among treatments within a matriline. Larvae in each treatment were raised in either 3 × 5 cm rectangular containers or 60 ml cups until second instar (container treatment was consistent within matrilines). They were transferred to 700 ml rectangular containers at the second instar and remained in these containers until they reached diapause, which usually occurs at the end of the fourth instar. Plant materials were replaced daily, and containers were cleaned every 1–2 days. Plants were field-collected from Glacial Heritage Preserve (46.87° N, 123.04°) weekly, with cut stems placed in water vials to prevent desiccation. Cut plants were stored under moist paper towels in a plastic cooler within a walk-in refrigerator (4 °C). Larvae were weighed to the nearest 0.1 mg at the third instar and again after reaching diapause.

Effects of diet on larval mass at third instar and again at diapause were tested using LMMs with matriline as a random effect. Significant effects of diet were followed by pairwise Tukey contrasts with the *emmeans*^40^ package.

### Objective 4: iridoid glycoside sequestration by larvae

We used gas chromatography to measure iridoid glycoside sequestration at diapause^41,42^. We used 1–2 larvae per treatment per matriline for these measurements. Caterpillars were frozen, then ground whole and extracted in 95% methanol for 24 h. The solid material was filtered out and methanol evaporated. After adding the internal standard, phenyl-β-D-glycopyranoside (PBG) at 0.500 mg/mL, each sample was partitioned with ether (3 times) to remove hydrophobic compounds. The ether layer was removed and the water layer (which contains the iridoid glycosides) was evaporated. The residue was suspended in 1.0 mL methanol, and a 100 µL aliquot removed for analysis. The methanol was evaporated and the remaining residue derivatized using Tri-Syl-Z (Thermo-Fisher Chemical Company) in pyridine before injection into an Agilent 7890A gas chromatograph equipped with a DB-1 column (30 m, 0.320 mm, 0.25 µm particle size) and using flame ionization detection. Amounts of four individual IG compounds, aucubin, catalpol, macfadienoside and methyl shanziside, were quantified using ChemStation B-03-01 software. Six samples were excluded due to labelling or processing issues.

Iridoid glycoside uptake was expressed both as the concentration and the total amount of iridoid glycosides in diapausing larvae. Each measure of uptake was tested using a LMM with matriline as a random effect. Significant effects of diet were followed by pairwise Tukey contrasts. Since caterpillars can contain multiple iridoid glycosides, we also tested if the composition of these compounds differed among diets using permutational multivariate ANOVA (PERMANOVA) in the R package *vegan*^43^. Values were relativized by the total IG amount in each larva so they expressed each compound as a proportion of the total. We used a Euclidean distance measure with 10,000 permutations.

### Objective 5: correlation of maternal preference, neonate preference, and larval growth

We tested if maternal preference correlated to that of their neonate offspring, and if larval mass gain on a given species could be predicted by maternal or sibling preference for that species (i.e., preference-performance relationships). In each case, we ran a separate linear model for each host plant species, first with maternal preference as a predictor and neonate preference of that matriline as a response, then with either maternal preference or neonate preference as a predictor and mean mass gain of that matriline as a response. We used mass data from larvae that ate bracts of *Castilleja* spp., rather than leaves, because this is what larvae generally feed on in the field.

## Results

### Objective 1: oviposition choice trials

**Figure 2:**
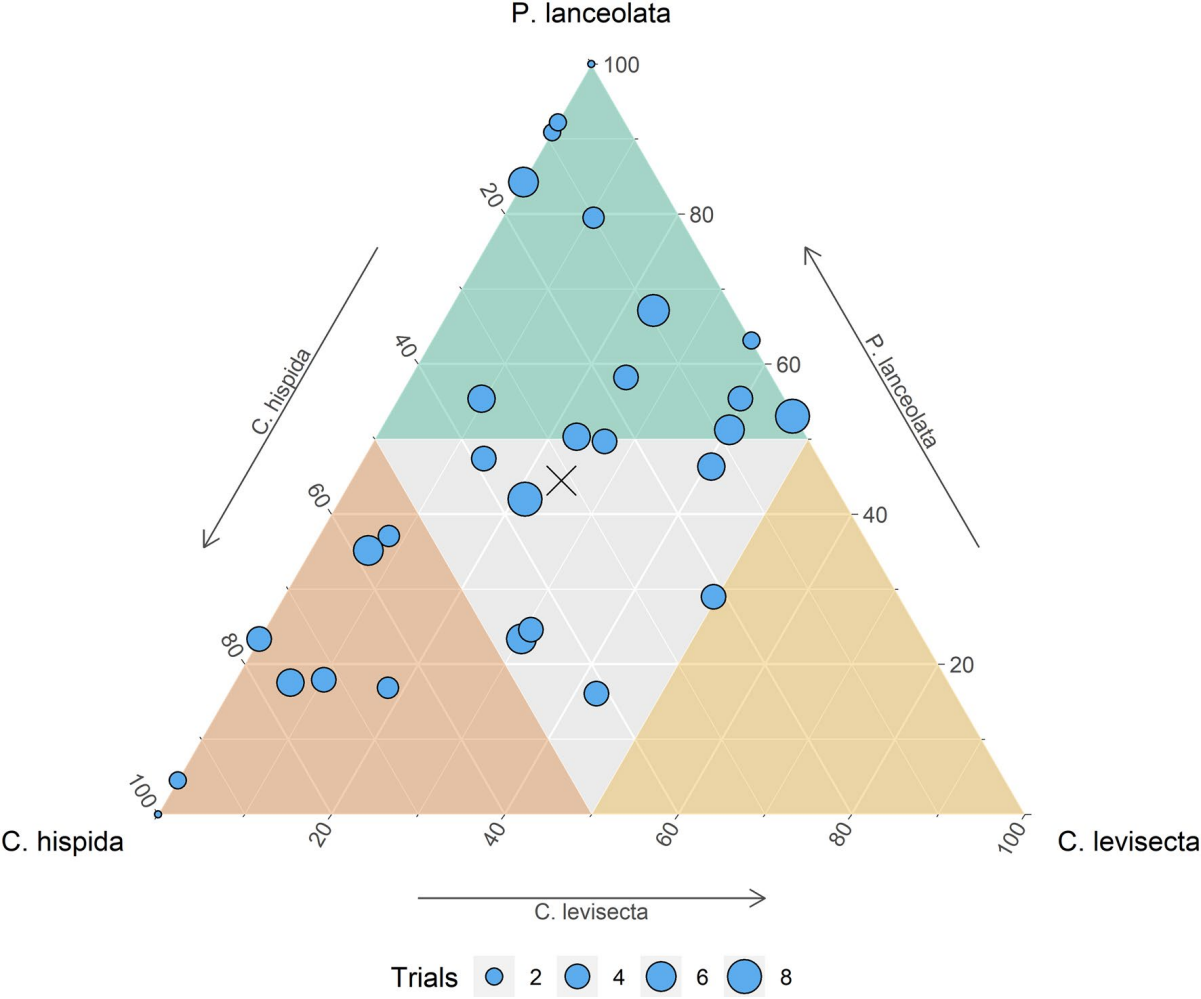
Ternary plot depicting oviposition preference of 29 Taylor’s checkerspot individuals among three host species. Each point represents an individual butterfly, and its position shows the overall proportion of eggs it allocated among the three species. Colors delineate areas in which more than half of an individual’s eggs are laid on that species. Point size is proportional to the number of trials each individual underwent. The population mean is shown by the ‘x’. Ternary plot generated using package *ggtern*^59^.

Population-level oviposition preference differed among the three host species (χ^2^_[2]_ = 11.7, p < 0.01). Of the 5417 eggs laid, 44% were placed on *Plantago,* 31% were on *C. hispida,* and 24% on *C. levisecta.* However, individual preference was quite variable. Some butterflies oviposited mostly on *P. lanceolata*, others mostly on *C. hispida,* and some used both or all three species (Fig. 2). Thirteen individuals allocated more than half their eggs to *Plantago*; eight allocated more than half to *C. hispida,* and none allocated more than half to *C. levisecta.* The remaining eight individuals apportioned their eggs more evenly among the three species. Eggs were laid in 312 clusters; 40% of these were on *Plantago,* 35% on *C. hispida,* and 25% on *C. levisecta* (this calculation omits rare instances when eggs were laid singly). Of the 29 butterflies, 16 laid their first cluster of eggs on *Plantago,* 10 on *C. hispida,* and 3 on *C. levisecta* (if they laid eggs on multiple species in the first trial, we considered the species that received more eggs to have been chosen)*.*

**Figure 3:**
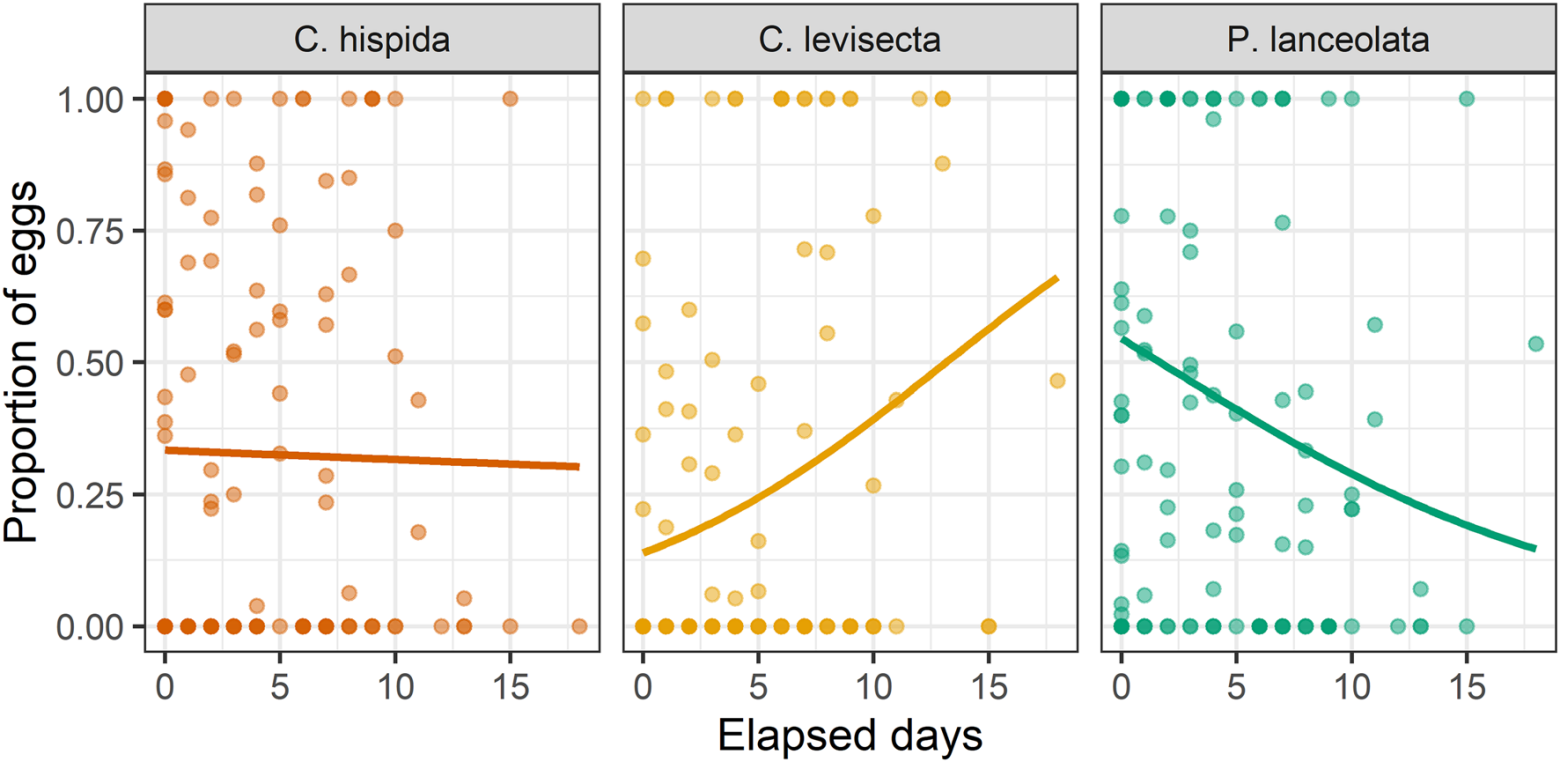
Oviposition preference changed over the course of the experiment. Each point on the plot is an individual trial (i.e., ~ 24 h period in which eggs were laid). Statistical results in Table 1.

Oviposition preference among the three plant species changed over time (Table 1, Fig. 3). Younger butterflies favored *P. lanceolata*, but preference for this species declined with time. Preference for *C. hispida* also declined with time, though not as steeply, while preference for *C. levisecta* increased.

**Table 1:** Results of GLMMs testing effects of time since first oviposition event on the eggs laid on each species as a proportion of the total.

Host species	Parameter	Value	SE	Z	p-value
*P. lanceolata*	Intercept	1.05	0.66	1.59	0.11
	Slope	− 0.31	0.02	− 14.48	< 0.01
*C. hispida*	Intercept	− 1.74	0.81	− 2.15	0.03
	Slope	− 0.09	0.02	− 4.05	< 0.01
*C. levisecta*	Intercept	− 11.97	1.63	− 7.36	< 0.01
	Slope	0.80	0.04	18.31	< 0.01

A separate model was run for each host species. Matriline and pot were included as random effects in all models.

### Objective 2: neonate choice trials

**Figure 4:**
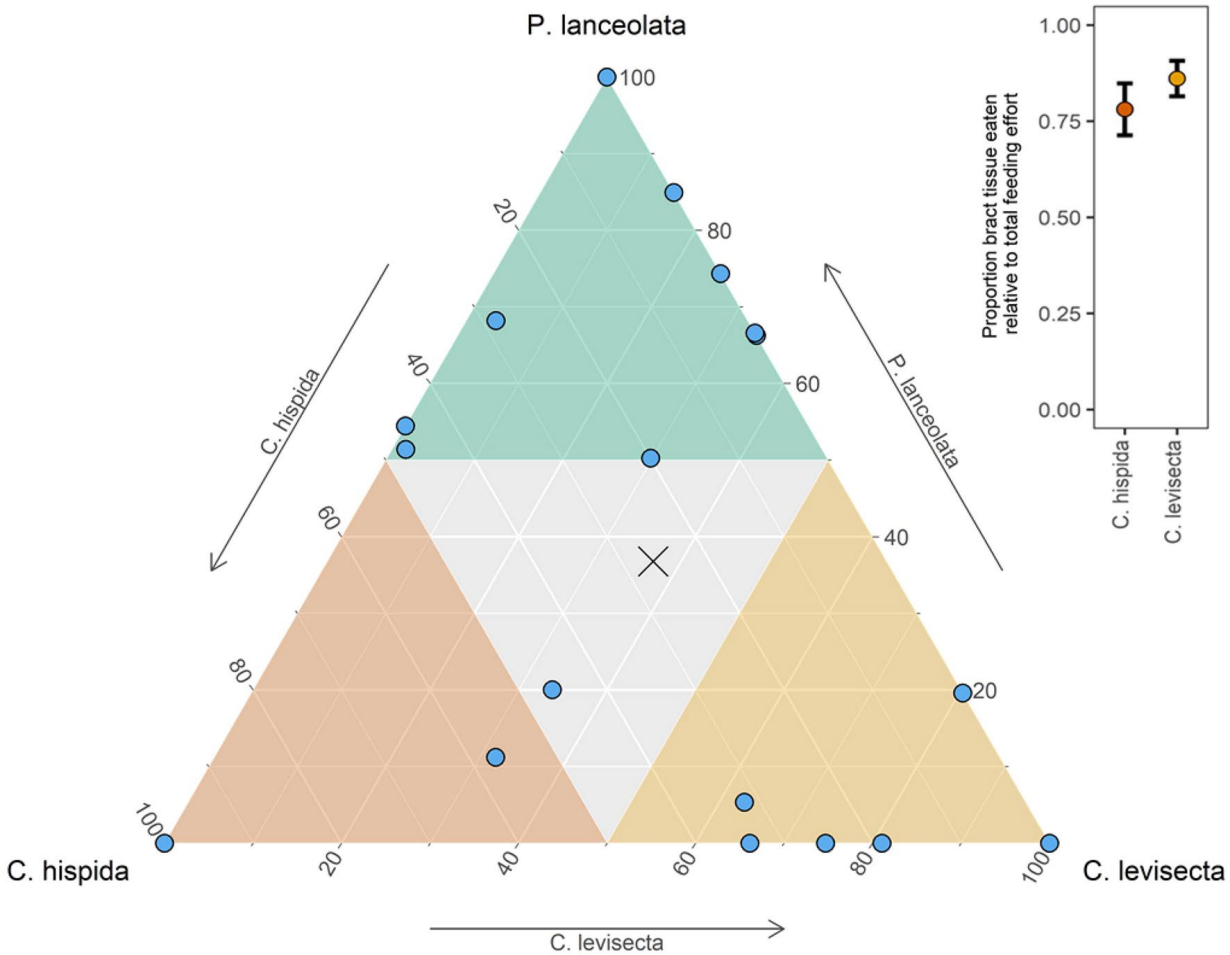
Ternary plot showing preference of 21 neonate sibling groups for leaf tissues of each host species (one choice trial per group, groups comprised of 3–6 individuals). Colors delineate instances in which more than half of the total leaf area eaten was that species. There are 2 overlapping points in each corner of the plot where neonates fed only on that species*.* The ‘x’ indicates population mean. Inset: when given the choice between bracts or leaves from the same stem of either *Castilleja* species, neonate larvae strongly preferred bracts. Points show mean proportion of leaf area eaten by each sibling group; brackets are ± 1 SEM.

We found no evidence of population-level neonate preference among the three host species (deviance = − 0.70, p = 0.70), although there was a non-significant trend for larvae to feed less on *C. hispida* than the other species. The average percent of total leaf area eaten by larval groups was 26% *C. hispida,* 37% *C. levisecta*, and 37% *P. lanceolata.* However, sibling groups from the various matrilines made diverse choices. Two groups fed only on *C. hispida,* two only on *C. levisecta,* and two only on *Plantago*; the remaining groups fed on two or all three species (Fig. 4). Thus, the population-level maternal oviposition preference toward *Plantago* and away from *C. levisecta* was not expressed by neonates.

Neonates strongly preferred bract over leaf tissue of both *Castilleja* species. When choosing among tissue types of *C. hispida*, neonates on average fed 78% on bract tissue and 22% on leaf (t_[20]_ =  − 4.15, p < 0.01). When choosing among tissue types of *C. levisecta*, they fed 86% on bracts and 14% on leaves (t_[20]_ =  − 7.83, p < 0.01) (Fig. 4).

### Objective 3: larval growth

**Figure 5:**
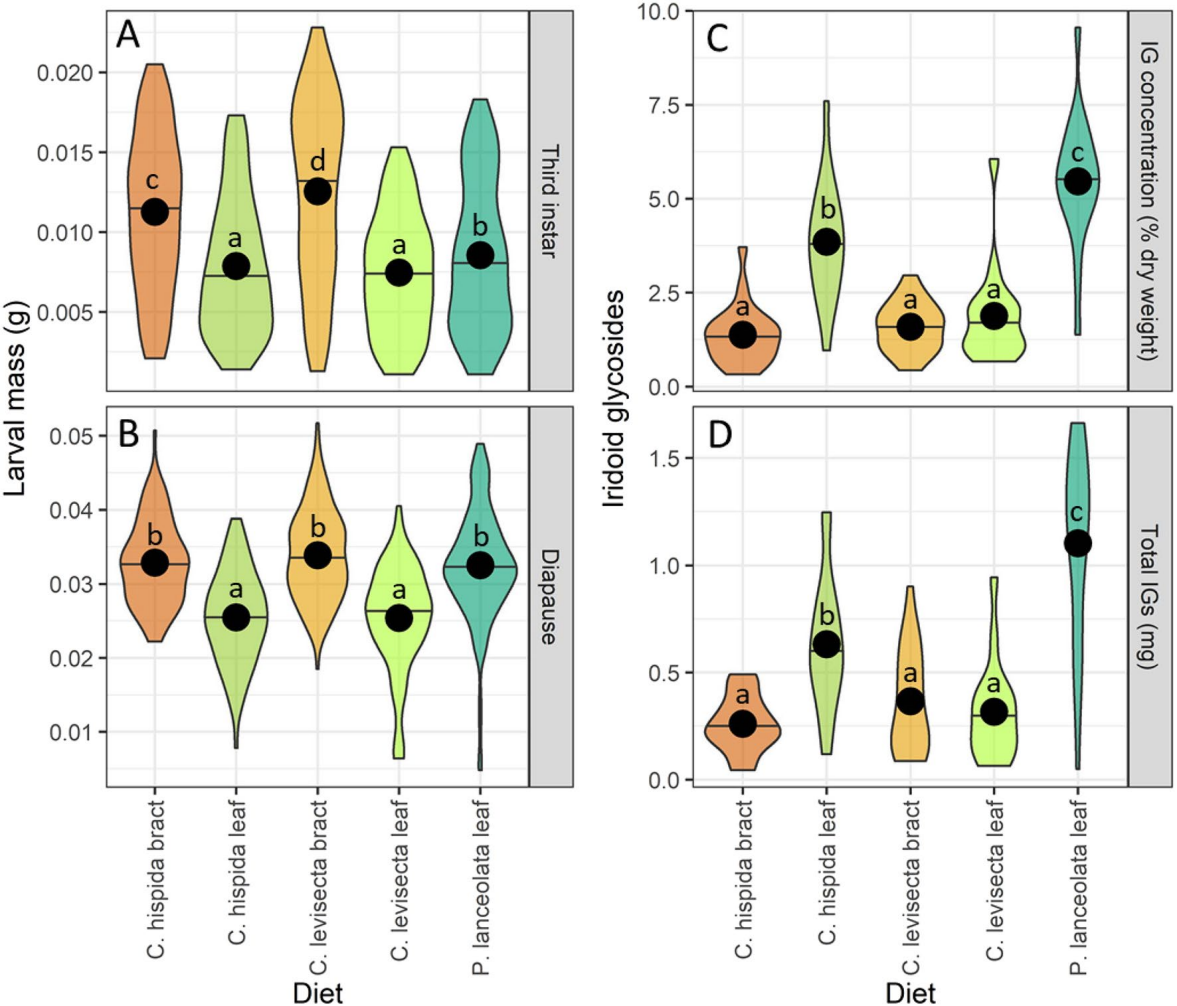
Mass gain and iridoid glycoside (IG) sequestration differed by treatment. Violin plots depict larval mass and IG sequestration responses to five no-choice feeding treatments. (**A**) Larval mass at third instar, approximately 2 weeks before diapause; (**B**) larval mass at diapause; (**C**) total IG concentration in larvae at diapause (% dry weight); (**D**) mass of IGs sequestered (mg). Horizontal lines represent medians, points represent means. Within each plot, treatments not sharing a letter differ significantly.

Mass gain differed strongly among feeding treatments both during third instar (F = 81.99, p < 0.01) and upon entering diapause (F = 54.09, p < 0.01). During third instar, larvae feeding on *C. levisecta* bracts were largest, followed by those feeding on *C. hispida* bracts and then those feeding on *Plantago*, while larvae feeding on leaves of either *Castilleja* species were smaller (Fig. 5A). At diapause, larvae that fed on *Plantago* or bracts of either *Castilleja* species were of similar mass and significantly larger than those that ate only *Castilleja* leaves (Fig. 5B).

### Objective 4: iridoid glycoside uptake

**Figure 6:**
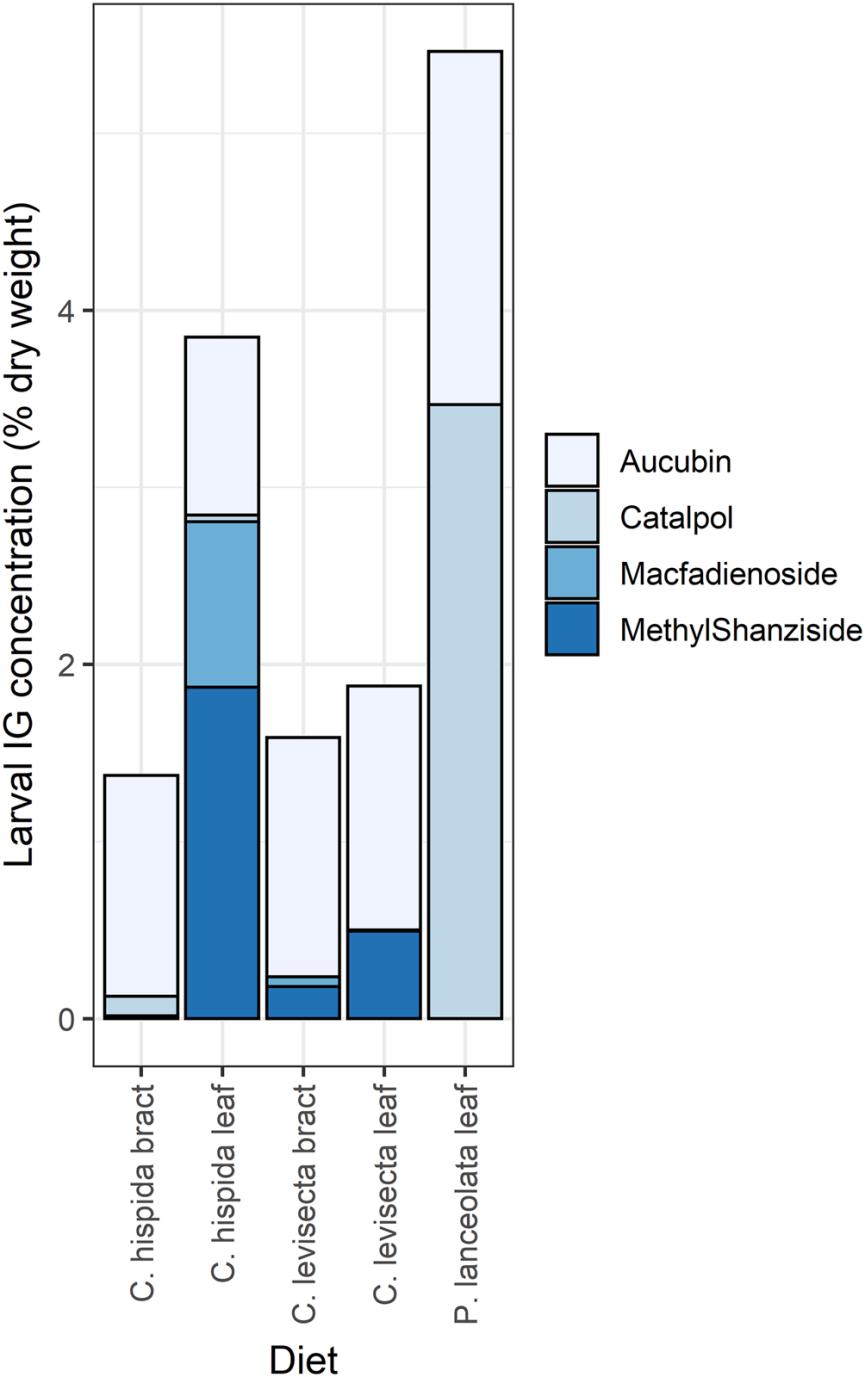
Composition of sequestered IG compounds differed strongly among treatments. Areas within each bar show the mean concentration of each of the four compounds found in larvae from that diet treatment, with one sample per matriline.

The total amounts of iridoid glycosides sequestered by larvae depended on their food plant, regardless of whether IGs were considered by concentration (F_[4,71]_ = 46.38, p < 0.01) or by mass (F_[4,71]_ = 32.76, p < 0.01). Uptake was highest in larvae that ate *Plantago*, intermediate in those that ate *C. hispida* leaves, and lowest in larvae fed other diets (Fig. 5C,D). We detected aucubin in every larva, along with up to three other iridoid glycosides depending on diet (Table 2, Fig. 6, PERMANOVA pseudo-F_[4,93]_ = 88.95, p < 0.01). Larvae that fed on *P. lanceolata* always contained catalpol. Larvae that fed on *C. hispida* sometimes contained catalpol but were more likely to contain macfadienoside and/or methyl shanziside. Larvae that fed on *C. levisecta* usually contained methyl shanziside, sometimes contained macfadienoside, and did not contain catalpol.

**Table 2:** Frequency of occurrence of iridoid glycoside compounds in larvae.

Diet	Aucubin	Catalpol	Macfadienoside	Methyl Shanziside
*C. hispida* bract	100 (22)	27 (6)	0 (0)	18 (4)
*C. hispida* leaf	100 (18)	11 (2)	94 (17)	100 (18)
*C. levisecta* bract	100 (22)	0 (0)	23 (5)	91 (20)
*C. levisecta* leaf	100 (16)	0 (0)	6 (1)	100 (16)
*P. lanceolata* leaf	100 (20)	100 (20)	0 (0)	0 (0)

Data are percent (number) of samples.

There were strong differences in iridoid glycoside uptake among larvae that ate different tissues within *Castilleja*. Most larvae that fed on *C. hispida* bracts contained only aucubin; a minority of these sequestered small amounts of catalpol and/or methyl shanziside, but none contained detectable amounts of macfadienoside. In contrast, those that ate leaves of the same species contained both aucubin and methyl shanziside, usually contained macfadienoside, and sometimes contained catalpol. Larvae that ate *C. levisecta* bracts always contained aucubin but usually also contained a small amount of methyl shanziside, occasionally contained macfadienoside, and never contained catalpol. Finally, those that ate *C. levisecta* leaves contained almost exclusively aucubin and methyl shanziside, with macfadienoside detected in only one individual and catalpol in none (Table 2, Fig. 6).

### Objective 5: correlation of maternal and neonate preference

Neonate preference for a given host did not correspond to the oviposition preference of their parent for that host (Table 3). Similarly, larval growth on each host was not predicted by either their mother’s or their siblings’ preference for that host (Table 4).

**Table 3:** Results of LMs testing whether feeding allocation by larvae correlated with maternal oviposition choice.

Host species	Model R^2^	Parameter	Value	SE	t value	p-value
*P. lanceolata*	0.04	Intercept	0.21	0.20	1.02	0.32
		Slope	0.33	0.39	0.84	0.41
*C. hispida*	0.03	Intercept	0.32	0.11	2.89	0.01
		Slope	− 0.22	0.31	− 0.71	0.49
*C. levisecta*	0.04	Intercept	0.27	0.14	1.96	0.07
		Slope	0.43	0.49	0.87	0.40

We found no evidence that maternal selection of a given species (i.e., the eggs laid on that species as a proportion of all eggs laid by that individual) correlated with offspring feeding preference (i.e., the amount of that species eaten by neonates as a proportion of their total feeding).

**Table 4:** Results of LMs testing whether mass gain by larvae on each host could be predicted by either maternal or sibling preference for that host.

Predictor	Host species	Model R^2^	Parameter	Value	SE	t value	p-value
Maternal preference	*P. lanceolata*	0.02	Intercept	0.02	0.00	6.53	< 0.01
			Slope	0.00	0.01	− 0.60	0.56
	*C. hispida*	0.08	Intercept	0.02	0.00	14.65	< 0.01
			Slope	0.00	0.00	1.20	0.25
	*C. levisecta*	0.03	Intercept	0.02	0.00	12.68	< 0.01
			Slope	0.00	0.01	0.76	0.46
Sibling preference	*P. lanceolata*	0.02	Intercept	0.02	0.00	10.68	< 0.01
			Slope	0.00	0.00	0.54	0.60
	*C. hispida*	0.18	Intercept	0.02	0.00	20.93	< 0.01
			Slope	− 0.01	0.00	− 1.95	0.07
	*C. levisecta*	0.02	Intercept	0.02	0.00	15.31	< 0.01
			Slope	0.00	0.00	0.62	0.54

## Discussion

The population we studied appears to be well-adapted to using *Plantago* but has retained preference for, and ability to perform on, its ancestral hosts. Butterflies readily laid eggs on all three host species, but they tended to favor *Plantago* and avoid *C. levisecta*. Larvae also developed similarly on all three species, with the main differences in larval mass at diapause being between those that ate leaves vs. bracts within the two *Castilleja* species. Iridoid glycosides were detectable in larvae fed all three hosts, although larvae tended to sequester greater amounts from *Plantago* and less from *C. levisecta* and IG composition varied.

### Causes and consequences of adopting a novel host

The ease with which checkerspots adopt *Plantago* and the number of taxa that have done so independently is remarkable^13–15^. A recent meta-analysis found that butterflies and moths generally perform poorly on novel exotic hosts compared to ancestral ones^44^, although there are certainly exceptions to this trend^45^. Checkerspots seem to illustrate ecological fitting, as they are broadly pre-adapted to *Plantago*^11,46^. Naïve larvae are often immediately able to develop on it, although in at least some cases adults initially reject it as an oviposition plant and preference for it must evolve^13^. A number of additional factors complement this close ecological fit and help explain why checkerspots repeatedly adopt *Plantago* and often perform well on it. *Plantago* can be very abundant in disturbed herbaceous habitats. In the case of Taylor’s checkerspot it is not only very easy to find but is also more phenologically available than native hosts because it senesces later^32^. Finally, the iridoid glycosides that *Plantago* produces, aucubin and catalpol, are already present in both *Castilleja* species investigated here, indicating that shifting from *Castilleja* to *Plantago* does not require metabolic adjustments as might be needed if a herbivore encountered a novel host containing new IG compounds. These two compounds are also common across other checkerspot hosts such as *Chelone glabra*^47^, the ancestral host of *E. phaeton* in Eastern North America. Many populations of this butterfly have also adopted *Plantago*^14^.

Although successful adoption of novel host plants can result in loss of adaptation to ancestral ones^4,12^, that was not the case here. The population we studied was almost entirely restricted to using *Plantago*, yet our results show that it has retained a breadth of oviposition and neonate feeding preferences. These findings contrast with outcomes for another *E. editha* population (subspecies *monoensis*) located about 900 km south of the one we studied, in Nevada, USA^12,13^. This population historically fed on *Collinsia parviflora* but switched to feeding entirely on *Plantago* over the course of three decades and lost the preference for its traditional host. The lost adaptation was this population’s downfall; it went extinct when *Plantago* became unavailable due to changes in agricultural management, despite *Collinsia* remaining available for oviposition^12^. There could be several plausible reasons why our study population did not lose adaptation to ancestral hosts. First, we do not know when our study population incorporated *Plantago* into its diet. This population could be derived partly or wholly from ancestors that adopted *Plantago* more than a century ago^33^, or the switch could be very recent (there could also be a history of repeated switches). If the switch occurred recently, loss of adaptation to *Castilleja* could still be in progress. Second, when the now-extinct Nevada population switched from *Collinsia* to *Plantago* it did so in the face of an evolutionary tradeoff in which butterflies experienced higher fecundity but slower development on *Plantago*, making for a poorer phenological fit with *Collinsia*^12^*.* We have not found evidence that Taylor’s checkerspot faces this sort of trade-off, so adding *Plantago* to its diet may not require losing adaptations to *Castilleja*. There is, however, some evidence that *Plantago* exerts selective pressure for larger clutch sizes, as larger sibling groups are more likely to survive to later instars on *Plantago* but not on *Castilleja*^32^.

### Variation in oviposition preference

Oviposition preference varied considerably among individual butterflies. While some strongly preferred either *Plantago* or *C. hispida*, many spread their effort more evenly among all three species (i.e., the 8 individuals in the center section of Fig. 2). Oviposition preference also varied within individual butterflies over time, as the proportion of eggs laid on *Plantago* and *C. hispida* decreased and the proportion laid on *C. levisecta* increased. There are multiple potential explanations for this. First, ovipositing *E. editha* become less selective over time^48^. However, if this was the only mechanism we would expect preference among the three species to become equal over time, and it did not. Second, butterflies may have innately avoided *C. levisecta* but learned and gradually accepted it over time—although individuals of other *E. editha* subspecies do not appear to learn regarding oviposition^49^. Finally, the suitability and attractiveness of plants may have shifted over the course of the study, as we moved plants daily between full-spectrum artificial light and sunlight during the two-week oviposition period. IGs, nitrogen, and water content of *Plantago* can change over short time intervals^50^, and relative attractiveness could also have changed over time as eggs were laid and leaves were removed, possibly inducing chemical responses in the plants. In light of these possibilities, we interpret oviposition patterns early in the study as being more indicative of preference in general. Field studies would be required to track oviposition over time and assess whether oviposition preference also varies in that context.

Our findings regarding oviposition contrast with previous work in which the same population appeared to prefer either *Castilleja* species over *Plantago*^34^. This difference could be attributable to variation in host plant characteristics, to the smaller number of lineages used in the previous study (5), or to differences in methodology, as the earlier study used abdomen-curling behavior as a signal of host plant acceptance^48^ rather than quantifying oviposition per se.

### Preference and performance

We did not find evidence for links between adult oviposition preference and neonate feeding choices, and neither of these forms of preference predicted larval performance. We would have interpreted a significant correlation between preference for, and performance on, a given host species as evidence that subsets of the population had evolved different host plant specializations, but this possibility was not supported. However, the ordering of oviposition preference (*Plantago* > *C. hispida* > *C. levisecta*) *did* match the frequency of interaction Taylor’s checkerspot from this region probably had with these species over the last several decades^29^, and their survival rates on them in the field^32^. In the field, both *Castilleja* species senesce before *Plantago* and early-instar larvae die in especially high numbers on *C. levisecta* as it begins to senesce in May and June^32^. In the present study we only fed non-senescent plants to larvae; the fact that they developed similarly on non-senescent *C. levisecta* as on other host species is more evidence that early senescence is the driving factor making *C. levisecta* less suitable in the field.

Neonate choices were variable, but neonates did not express strong population-level preference among host species, and when they selected a certain species this did not correlate to better performance on that species by their siblings. However, they expressed strong preference between tissue types in *Castilleja,* choosing bracts over leaves. This choice appears adaptive since eating bracts resulted in higher mass gain (Fig. 5A,B). Neonates must choose which tissues to eat within a plant but are rarely required to choose among plant species (they typically remain on the natal plant until instar 2 or 3); this aligns with our finding that they strongly preferred the higher-quality tissue type within *Castilleja* but did not have strong preferences among species.

In the field, eggs are typically laid on leaves toward the bottom of the stem, and larvae move to the plant apex by the second instar to feed on bract and flower tissues (although they still eat some lower leaves as well; Haan pers. obs.). It is counterintuitive that adults lay eggs on a tissue type that larvae avoid and that is nutritionally inferior, but they could be choosing oviposition sites to hide eggs from enemies, based on within-plant differences in secondary chemistry^22^ or based on abiotic conditions required for egg development.

Larval growth varied among diets and over time. *Castilleja* bracts gave an early advantage to larvae, but by diapause, larvae that fed on *Plantago* had caught up and were similar in size (Fig. 5A,B). This may have occurred because *Castilleja* bracts are soft and contain fewer trichomes (Haan pers. obs.), making them easy for early instars to eat quickly. We also noticed that larvae in the first and second instars ate entire *Castilleja* bracts but skeletonized the thicker *Plantago* leaves. However, during the third and fourth instars larvae were able to eat entire *Plantago* leaves rather than skeletonizing them and quickly made up for lost growth.

### Iridoid glycoside uptake

In general, overall IG concentrations in larvae were similar to those in previous studies of related taxa at similar ontogenetic stages^41,51,52^. Concentrations were highest when larvae ate *Plantago* or *C. hispida* leaves and lower if they ate *C. hispida* bracts or either tissue type of *C. levisecta*. This is consistent with data from individuals that fed on these species in a field setting: those that ate *C. levisecta* sequestered substantially less than those that ate the other species^53^. Since sequestered IGs are thought to deter natural enemies^54,55^, the reduced ability to sequester from *C. levisecta* could increase predation risk. Our results also indicate that intra-specific and intra-individual variation within *C. hispida* is important with respect to larval performance: larvae that fed only on *C. hispida* bracts grew larger but had lower IG concentrations than those that fed only on *C. hispida* leaves. In a field setting, larvae would be mobile and able to forage across both tissue types, perhaps optimizing larval performance and sequestration.

Larvae carried distinct chemical fingerprints depending on their diet. If they ate *Plantago,* they contained aucubin and catalpol (the two IGs found in *Plantago*), while if they ate *Castilleja* they contained up to two additional compounds, macfadienoside and methyl shanziside (found in both *Castilleja* species). There were striking differences between larvae that ate different *C. hispida* tissues; they sequestered large amounts of macfadienoside when they ate leaves, but none if they ate bracts (Fig. 6). IGs can be spatially segregated among *Castilleja* tissues; for example, within *Castilleja integra,* macfadienoside is abundant throughout the plant but especially in leaves, methyl shanziside is much more common in leaves than in bracts or flowers, and catalpol dominates flowers^35^.

In previous work we found that greenhouse-grown *C. levisecta* produced largely aucubin, and *E. editha* larvae that ate it contained both aucubin and catalpol^52^; this contrasts with our findings here. These differences in plant chemistry are likely because plants in the previous study were grown in the greenhouse, whereas plants used in the present study were field collected. Greenhouse grown plants are often lower in secondary compounds than those grown outside or collected from the wild^56^. It is also not known if the four compounds derived from *Castilleja* have different costs or benefits to specialist herbivores. Previous work with another IG specialist, the buckeye, *Junonia coenia* (Nymphalidae)*,* showed that aucubin and catalpol have positive synergistic effects on survival, growth, and sequestration rates but negative effects on immune response^57,58^. The specific effects of macfadienoside and methyl shanziside on herbivores and their enemies have not been investigated. Given that amounts of these compounds in larvae differ sharply depending on host species and on tissue type eaten within *Castilleja* spp., they could have important implications for herbivore immune function and/or interactions with higher trophic levels.

### Implications for conservation efforts

Taylor’s checkerspot has been reduced to a very small number of populations that mostly rely on *Plantago*, and our data show that the population we studied prefers it and performs well on it. However, we also found that a preference for, and ability to develop on, other hosts continues to persist. While we did not directly assess genetic diversity, it seems that this relict population is well-suited for reintroductions to sites where host plant availability differs from the source site.

Our results cast further doubt on the suitability of *C. levisecta* as a primary host plant for Taylor’s checkerspot in Washington lowlands*.* Although it was almost certainly an ancestral host, it is rare and may not have been used as frequently, at least during the last several decades^29^. Adults will oviposit on it but tend to favor other species and, although larvae do not innately avoid it, they have lower survival rates on it in the field^32^ and sequester relatively lower concentrations of IGs from it. However, since *C. levisecta* does not appear to be especially attractive to ovipositing butterflies and larvae can successfully develop on it if needed, it could be acceptable as a secondary host plant and does not appear to pose undue risk to recovery efforts. Furthermore, given that *E. editha* host plant affiliations evolve rapidly, populations that are reintroduced where *C. levisecta* is the main available host might readily adapt to it.

Taylor’s checkerspot host affiliations are not static; the patterns we documented in this study are likely to change in the future both for this population and for the reintroduced ones that are derived from it. While there may be uncertainty about relative suitability of host resources, the diversity in preference and performance we documented here is probably an asset. Management for Taylor’s checkerspot should aim to maintain many populations using diverse host resources. Population-level differences in host specialization, if they occur, will help buffer against future changes and make recovery efforts more likely to succeed over the long term.

## Data Availability

Data from this study were submitted to Dryad. https://doi.org/10.5061/dryad.612jm642h.
